# Pressure Sensor System for Customized Scoliosis Braces

**DOI:** 10.3390/s21041153

**Published:** 2021-02-06

**Authors:** Franz Konstantin Fuss, Asliza Ahmad, Adin Ming Tan, Rizal Razman, Yehuda Weizman

**Affiliations:** 1 Smart Products Engineering Program, Centre for Design Innovation, Swinburne University of Technology, Melbourne, VIC 3122, Australia; amtan@swin.edu.au (A.M.T.); uweizman@swin.edu.au (Y.W.); 2 Centre for Applied Biomechanics, Department of Biomedical Engineering, Faculty of Engineering, University of Malaya, Kuala Lumpur 50603, Malaysia; asliza90@gmail.com; 3 Centre for Sport & Exercise Sciences, University of Malaya, Kuala Lumpur 50603, Malaysia; rizal@um.edu.my

**Keywords:** pressure sensor, scoliosis brace, foam properties, hysteresis, calibration, cost-effective development

## Abstract

Hard-shell thoracolumbar sacral orthoses (TLSOs) are used for treating idiopathic scoliosis, a deformation of the spine with a sideways curvature. The pressure required inside the TLSO for ideal corrective results remains unclear. Retrofitting TLSOs with commercially available pressure measurement systems is expensive and can only be performed in a laboratory. The aim of this study was to develop a cost-effective but accurate pressure sensor system for TLSOs. The sensor was built from a piezoresistive polymer, placed between two closed-cell foam liners, and evaluated with a material testing machine. Because foams are energy absorbers, the pressure-conductance curve was affected by hysteresis. The sensor was calibrated on a force plate with the transitions from loading to unloading used to establish the calibration curve. The root mean square error was 12% on average within the required pressure range of 0.01–0.13 MPa. The sensor reacted to the changing pressure during breathing and different activities when tested underneath a chest belt at different tensions. The peak pressure reached 0.135 MPa. The sensor was further tested inside the scoliosis brace during different activities. The measured pressure was 0.014–0.124 MPa. The results from this study enable cheaper and mobile systems to be used for clinical studies on the comfort and pressure of braces during daily activities.

## 1. Introduction

Idiopathic scoliosis (IS) is a condition of deformation of the human spine, affected by sideward deflection (bending) and torsion, with unclear etiology.

According to Weinstein et al. [1], 1–3% of children aged 10–16 years are affected by IS; according to Konieczny et al. [2], 0.5–5.2% of the whole population is affected by this condition. In female children, the prevalence and the deflection angle are greater, and the female-to-male ratio increases as the age increases [2].

The sideward deflection angle, referred to as the Cobb angle, is used for diagnostic purposes and therapeutic decision making. Generally, Cobb angles ranging from 20° to 45° are treated with a thoracolumbar sacral orthosis (TLSO) or brace to stabilize the spine and prevent further increment of the angle. IS, if left untreated, leads to various problems in later life, such as disturbed pulmonary function, back pain, or even death [3].

Many studies have reported good outcomes from the usage of the TLSO to halt curve progression and improve the quality of life [4,5]. TLSO-type braces, such as Boston, Chêneau, and Milwaukee braces, generally work on three main support points depending on the location of the curves. The main support points apply corrective pressure to the scoliotic curve introduced to the patient’s trunk through the brace. The three main support points are the apex of the thoracic or thoraco-lumbar curve and, on the opposite side, the sub-axillary and pelvic supports [6].

Although previous studies have reported on the positive effect of TLSOs for IS patients, the main mechanism of action between the patient trunk and the brace is still unclear. Several studies have been undertaken on pressure measurements to identify the magnitude and forces inside the braces. Wong and Evans [7] measured the interface pressure between the patient’s body and a Milwaukee brace with a commercially available electrohydraulic pressure-sensing system; van den Hout et al. [8] used capacitive sensors (Pedar, Novel, Munich, Germany) in a Boston brace; and Pham et al. [9] and Ahmad et al. [10] used piezoresistive sensors (TekScan Inc., Boston, MA, USA) for pressure measurements in a Chêneau brace. The pressure range obtained was large, from approximately 0.001 MPa (lowest value shown by Wong and Evans, 1998) to 0.112 MPa (highest average reported by [10]). Although these studies used different measurement systems and different types of TLSO, the data suggest that the ideal pressure level for comfort and an optimal therapeutic outcome remains unclear.

The common choice of pressure sensors in the medical field are piezoresistive or capacitive sensors. Piezoresistive sensors are relatively cheap but nonetheless sufficiently accurate for measurement of pressure between skin and garments or shoes. For example, Belbasis and Fuss [11,12] and Belbasis et al. [13,14] used customized piezoresistive polymers to measure the pressure between the skin and a compression garment for assessment of muscle activity and fatigue (mechanomyography, force-myography (FMG)). Other FMG applications with piezoresistive sensors, encompassing off-the-shelf force sensing resistive (FSR) sensors such as single, multiple, and matrix sensors, have been reported for different applications [15,16,17,18,19,20].

Weizman and Fuss [21] and Fuss et al. [22] used piezoresistive vinyl to measure the pressure between the instep and a soccer boot, measuring the kick force and localizing the sweet-spot. Tan et al. [23] developed a low-cost smart insole from piezoresistive polymers that were later replaced by screen-printed sensors [24,25]. Within scoliosis braces, long-term pressure measurements have yet to be made. Moreover, existing commercially available pressure sensor systems are too expensive and user-unfriendly for long-term pressure monitoring. The lack of user-friendliness is related to hardware that is difficult to wear (such as bulky hardware including connectors and wires on the patient’s body) and non-customized software. Furthermore, there is no consensus on the required magnitude of corrective pressure for ideal therapeutic results, simply because of the two aforementioned problems.

It was, therefore, the aim of this study to develop and test a low-cost, but accurate pressure sensing system for scoliosis braces by addressing the challenges related to pressure sensors for medical applications. These challenges refer to sensor design, evaluation, calibration, validation, and costing, in addition to testing of special events (e.g., breathing), and the final application inside the brace.

## 2. Materials and Methods

### 2.1. Rationale of Study Design and Methodology

**Figure 1:**
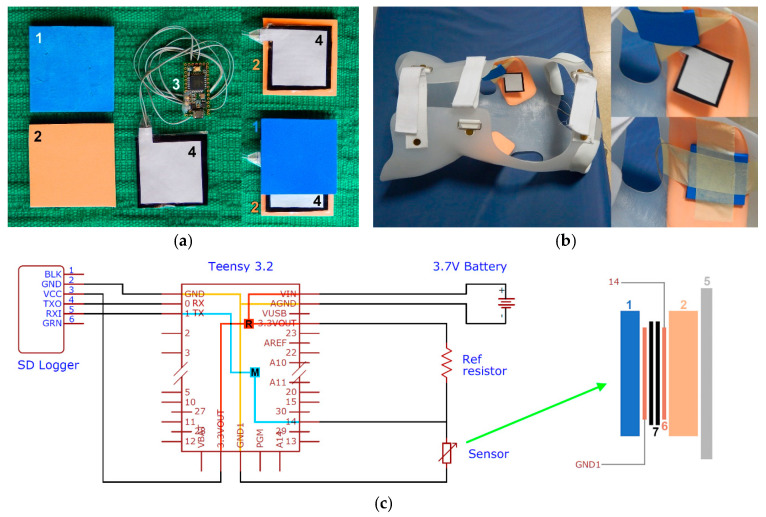
(**a**) Sensor system: 1st column: 1: softer foam (blue); 2: denser foam (pink); 2nd column: 3: Teensy and microcontroller; 4: sensor (black: piezoresistive polymer, white: backside of a copper electrode); 3rd column: assembly process: sensor 4 placed on denser pink foam 2, and softer blue foam 1 placed on sensor 4 (in the final assembly, both foams are superimposed); (**b**) sensor placed inside a Chêneau brace and taped to the inner wall of the brace; (**c**) circuit and electronics diagram, and sensor cross section; R: regulator (3.7 V (VIN) to 3.3 V (3.3VOUT)); M: microcontroller (analog drop voltage signal from port 14 converted to digital ASCII data recorded by the SD logger (Secure Digital memory card)); 5: hard shell of brace; 6: copper electrode; 7: piezoresistive sensor (two layers); GND: ground; TX, RX: transmitting and receiving ports.

This study was divided into several steps, each of which was required for solving specific problems or for conducting different tests. This approach spans the research and development process from sensor design, to measuring the pressure inside a scoliosis brace (Figure 1a,b). The sensor was developed from experience in similar sensors of previous projects [11,12,13,14,25,26,27]. Due to the foam padding inside the scoliosis brace and the knowledge [25] of how materials placed next to a piezoresistive sensor affect their electrical properties, the two foams involved had to be tested and characterized mechanically. Subsequently, the sensor behavior (pressure versus conductance) was evaluated in four configurations (sensor tested without foams, with one foam or the other, and sandwiched between both foams) to understand the influence of the foams on the sensors from an electro-mechanical perspective (unequal loading effect on conductance). This step led to the selection of the four configurations, which were used in all subsequent experiments. The next step was to calibrate the sensor over a wide pressure range and test its accuracy. After calibration, the sensor was tested on the human body under different belt stiffnesses and tensions and in various activities, to understand the practical sensitivity, repeatability, and magnitude of the pressure data, and determine whether the sensor is suitable and ready for a clinical test. Subsequently, the sensor was tested inside the scoliosis brace of two patients (by taping the sensor to the inner side of the brace at the high-pressure areas; Figure 1b), according to a test protocol used previously [10].

### 2.2. Sensor Design and Assembly

The sensor material selected for pressure measurements was an electrostatic carbon-infused polymer (Velostat, 3M, St. Paul, MN, USA) with piezo-resistive properties, applied in previous research projects [11,12,13,14,26,27], because of its accuracy and cost-effectivity. The polymer matrix of this material is made of polyolefin [28], and the electrical resistivity of this material in the unloaded state is approximately 23 kΩm. Two 0.1 mm thick layers of this material were found to deliver the most accurate results [12,26]. The size of the sensor was 45 × 45 mm, sandwiched between two copper foil electrodes (40 × 40 mm; Figure 1a). The effective area of the sensor was therefore 1600 mm^2^. The sensor was placed between the two foam layers (Figure 1a), foam A of 3.17 mm thickness, and foam B of 6.15 mm. Foam A was located next to the skin if used in a scoliosis brace, and foam B was the liner of the hard polymer shell of the brace (Figure 1b). At this stage, it should be noted that the pressure sensor was never in permanent contact with the patients; rather, it was only intermittently and indirectly in contact with the patient when wearing the brace, because the pressure sensor system was taped to the inner side of the brace (Figure 1b) at the high-pressure points. There were two reasons for this. First, if the sensor is permanently attached to the patient’s skin, then the electronics unit has to also be connected to the patient with a cable and tape (a non-tethered version, i.e., a wireless version, requires a data transmitting unit, e.g., Bluetooth, which is located on the PCB (printed circuit board) of the electronics unit and not on the sensor system, so that the electronics unit and the sensor system remain connected by a cable). It is not advisable to have the electronics on the body of the patient, not only for biomedical safety requirements, but also for hygienic reasons; when patients shower, they must remove the electronics unit and sensor system, and then place it again on exactly the same spot. These problems are prevented when attaching the sensor permanently to the inner side of the brace (Figure 1b), and the electronics unit to its outer side. Secondly, it is imperative to measure the pressure at the points of the highest pressure, because this is where the therapeutic pressure is administered. These high-pressure points are evidently invisible on the patient’s skin, but highly apparent on the inner side of the brace, where the brace is waisted. Attaching the sensor to the inner side of the brace (Figure 1b) guarantees that the pressure is always measured at the same high-pressure location.

The pressure sensor was connected to the analogue input of a programmable microcontroller (Teensy 3.2, 32-bit ARM Cortex-M4 72 MHz CPU, PJRC, Sherwood, OR, USA), powered by a chargeable battery (3.7 V, Polymer Lithium Ion), and in series with a 47 Ω reference resistor before connecting to ground. The change in drop voltage with pressure was recorded on a data logger (OpenLog, SparkFun Electronics, Niwot, CO, USA) at a sampling frequency of 10 Hz. The total cost of the electronics amounted to AUD 90.

### 2.3. Foam Properties

Two closed-cell foams were used to sandwich the sensor, and are typically inner liners that cushion the prosthetics and orthotics. The inner foam layer (foam A: closed-cell ethylene-vinyl acetate copolymer foam, color: blue) served as the comfort layer in contact with the patient, whereas the outer layer (foam B: Pe-Lite, closed-cell polyethylene foam, color: pink), in contact with the inner side of the brace, was a standard liner of prosthetics and orthotics, placed at the high-pressure areas inside the brace. The combined thickness of the foams and the sensor system was not to exceed 10 mm due to the restricted design space for foam padding inside the brace.

Their properties were determined with a material testing machine (Instron 5967, Instron, Norwood, MA, USA, with a 30 kN load cell). Five samples of foams A and B were compressed at a deflection rate of 0.032 s^−1^ up to 2500 and 5000 N, respectively. The displacement and force data were converted to strain (*ε*) and stress (*σ*). The tangent moduli (*E*) were calculated from the strain derivative of the stress, and the energy absorbed per unit volume (*W*) was calculated from the stress integral with strain. Subsequently, the ratio of *W* to *σ* [29] was calculated, and determines a dimensionless index of the greatest amount of energy absorbed per unit stress. There are standards for foam testing, such as ISO 3386-1 [30] and ASTM D3574-C [31], but they compress the foams only up to 40% and 50% of strain, respectively, and report the CV40 and CV50, respectively, i.e., the compression value (stress or pressure) at up to 40% and 50% of strain. A single stress datum fails to characterize a cellular structure in its entirety, and therefore the test standards were replaced by reporting continuous mechanical foam data.

### 2.4. Sensor Evaluation

The sensor evaluation enhanced understanding of the sensor’s behavior when used with foams. It is known from previous research [25] that viscous materials placed on piezoresistive sensors increase the intrinsic sensor viscosity. The latter becomes apparent from time-dependent effects, such as stress relaxation, creep, and hysteresis behavior. Foams, in addition to being viscous, encompass other mechanisms of energy absorption, such as buckling elements and pneumatic springs [29]. It is therefore expected that any additional foam layer will increase the hysteresis of the loading–unloading cycle used for calibration. To verify this behavior, the sensor was calibrated under 4 different configurations:

1. sensor without any foam layer;
2. sensor placed on foam A;
3. sensor placed on foam B;
4. sensor sandwiched between foams A and B (configuration used inside a scoliosis brace).

For any of these configurations, the sensor was compressed with an Instron material testing machine over 5 loading/unloading cycles at a crosshead speed of 0.02 mm/s, with a triangular displacement function, up to a pressure of 0.306–0.31 MPa. The drop-voltage data, measured across the reference resistor including the timestamp, were collected with a Teensy at 10 Hz. The force, displacement, and time data were collected by the Instron at a data sampling frequency of 10 times that of the Teensy. The higher recording frequency allowed the corresponding force data to be assigned to the timestamp of the Teensy, after alignment of the 5 peaks of both signals (force and voltage, based on their corresponding time data) in MATLAB (MATLAB 2018a, Mathworks, Natick, MA, USA). Subsequently, the force, converted to pressure, was plotted against the conductance of the sensor. The differences between the pressure–conductance plots in all 4 different configurations were inspected.

### 2.5. Sensor Calibration and Validation

The calibration method was selected after considering the following shortcomings:

1 It was already known from the sensor evaluation exercise that configuration 4 showed the greatest hysteresis; it was therefore decided to use only peak data to establish the calibration curve.
2 To prevent any method-related influence, it was decided to calibrate and validate the sensor with exactly the same method, using a force plate (Kistler, 9260AA6, Winterthur, Switzerland) as a gold standard. The force readings were converted to pressures because the area applied to the sensor was constant.
3 Sensors used in scoliosis braces do not undergo fast and repetitive high-frequency changes of pressure; the only cyclic pressure change would be the respiration frequency and, other than this, the pressure data would be more static. Accordingly, the sensor was loaded manually on the force plate with impulses ranging between sinusoidal and trapezoidal shapes of intended durations between 2 and 8 s (actually 1.7–8.7 s during the experiments).

A peak detecting algorithm was used to identify the signal fluctuations (corresponding pressure and conductance data) at the peak regions or plateau regions of the pressure signal. For each loading/unloading event of the sensor, the peak pressures and the corresponding conductance were averaged to a single pressure and conductance datum. These averaged peak pressure data were plotted against the corresponding conductance, and a polynomial curve was fitted to the data to establish the calibration curve. The latter was subsequently used to convert the conductance of all peak pressure data. Based on the residuals (differential between the measured force from the force plate data converted to pressure relative to the sensor’s effective area, and the calculated pressure from the sensor’s conductance and the calibration function) expressed as a percentage of the pressure obtained from the force plate, the RMSE% (root mean square error as a percentage [24]) was calculated. Bland–Altman plots were used as an additional validation tool.

### 2.6. Pressure Measurements

The sensor was tested using sensor configuration 4. The configuration was used for all pressure measurements because of the sensor evaluation method, explained in Section 3.2 below. The sensor was again sandwiched between foams A and B, with a hard polymer plate, used for scoliosis braces, on the outer side. The sensor configuration was placed between a strap (leather belt or elastic band (A300, Polar, Kempele, Finland)) and the skin on the right side of the chest at different belt tensions for several minutes. The activities performed during the tests were: walking; climbing and descending stairs; deep thoracic breathing (at the inspirium); abdominal breathing; and lying on bed in different positions. The time sequence of the different activities was recorded, and the test person applied artificial pressure spikes to the sensor manually between the activities for identification purposes. The conductance data were converted to pressure using the calibration curve obtained from the sensor calibration exercise. The data were recorded at a sampling frequency of 1 Hz.

The following tests were self-conducted by one of the authors (F.K.F.) of this paper:

Test 1: the pressure was applied with a leather belt, with the buckle at the 3rd and then at the 4th hole (from the end of the belt);
Test 2: a softer, elastic belt (A300, Polar, Kempele, Finland);
Test 3: increasing the pressure with the elastic belt (A300, Polar, Kempele, Finland);
Test 4: with the leather belt at the 3rd hole.

The rationale of these 4 tests was to understand the pressure dynamics of a sensor sandwiched between a compressive device (belt in this case) and the human body. These tests are analogous to those carried out by Belbasis and Fuss [11,12] and Belbasis et al. [13,14], who used a compression garment instead of a belt to correlate the muscle force to the pressure produced by the bulging muscle (force myography). In contrast to previous studies [11,12,13,14], the present tests allowed sensor data to be obtained of (a) changes in pressure when breathing (force thoracography) and their pressure magnitudes at different belt stiffnesses and compressions; (b) pressure produced by abdominal and thoracic breathing; (c) activity-related pressure changes; and (d) external pressure influences, such as lying directly on the sensor. The overarching goal of the 4 tests was to understand whether the sensing range of the sensor system, as per its calibration curve, was appropriate to measure these pressures.

Ethics approval for these tests was not required because these tests were conducted via self-testing to obtain sensor data rather than participant-related data (according to the ethics committee of Swinburne University).

The sensor (configuration 4) was tested inside the Chêneau scoliosis brace (Figure 1b) of two patients with Adolescent Idiopathic Scoliosis from the Outpatient Scoliosis Clinic, University Malaya Medical Centre (UMMC), Kuala Lumpur, Malaysia. The sensor was placed at the levels of the thoracic and thoracolumbar curves. For this purpose, the pressure sensor system was taped to the inner side of the brace (Figure 1b). The participants had to perform nine different activities (standing, maximum inspiration, maximum expiration, walking, sitting, supine position/lying on the back, lying on the right side, lying on the left side, prone position/lying on the abdomen), i.e., the positions that were investigated by Pham et al. [9] and Ahmad et al. [10], and partially by van den Hout et al. [8]. The central 50 pressure data points per position were used for statistical analysis. The pressure data at the different positions were compared with a one-way ANOVA test and the significant differences were determined with the following post-hoc tests: Tukey, Scheffé, Bonferroni, and Holm. The pressure data at the thoracic and thoraco-lumbar curve were compared with the independent samples t-test. Written informed consent was obtained from the parents and assent from the patients to participate in the study was obtained prior to enrolment. Ethical approval was obtained from the UMMC Medical Ethics Review Committee.

## 3. Results

### 3.1. Foam Properties

**Figure 2:**
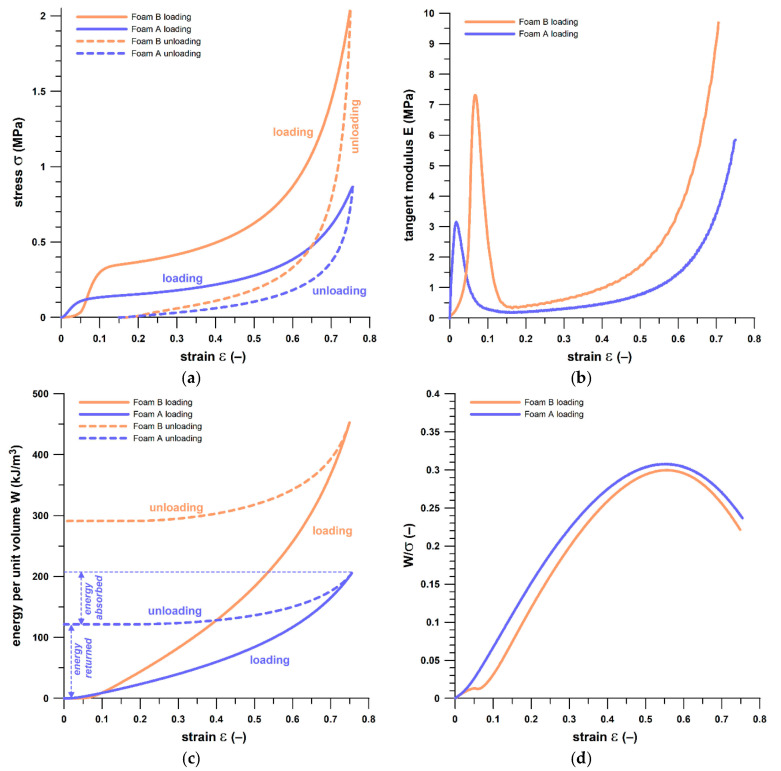
(**a**) Stress vs. strain; (**b**) tangent modulus vs. strain curves (loading segment) (**c**) energy per unit volume vs. strain; (**d**) ratio of energy per unit volume to stress vs. strain (loading segment) of foams A (blue) and B (pink).

Foam B was twice as thick as foam A. Foam B was subjected to twice the force (5 kN) of foam A (2.5 kN), and the strain at the maximal forces was similar, namely 74% and 76%, respectively (Figure 2a). Both foams exhibited the typical hysteresis behavior expected from energy absorbing materials, in which the area inside the hysteresis corresponds to the energy (per unit volume) absorbed. Foam B absorbed slightly less energy (35%) than foam A (41%) under the given test conditions. The energy absorption is shown in Figure 2c. Foam B was twice as stiff as foam A at the linear elastic segment, and 2.5 times stiffer at the collapse plateau (Figure 2b). Both foams exhibited the same maximum *W*/*σ* ratio (approximately 0.3), the same *W*/*σ* trend (close parallel curves; Figure 2d), and same strain (55–56%) at maximum *W*/*σ*. The matching *W*/*σ* data reveal that internal structures of both foams were geometrically similar. The foam properties and the experimental conditions are summarized in Table 1 and Figure 2.

A thorough characterization of both foams is essential because it is known [25] that any material exhibiting time-dependent effects and placed next to a piezoresistive sensor affects the sensor properties, and particularly the pressure–conductance relationship (cf. Section 3.2).

### 3.2. Sensor Evaluation

**Figure 3:**
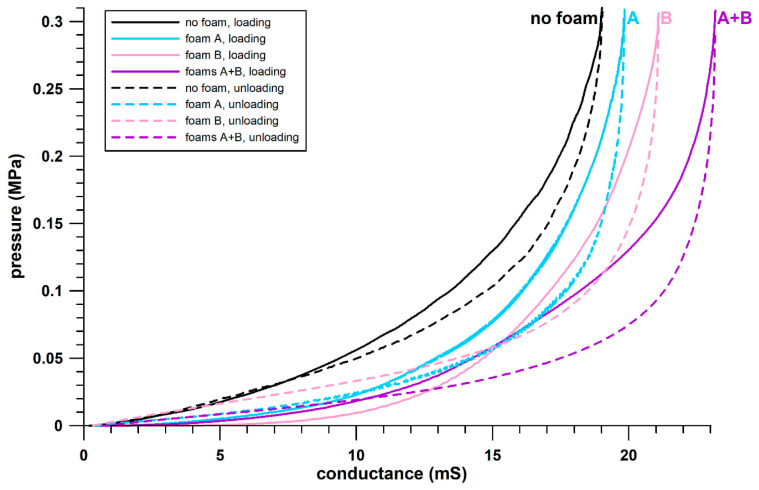
Pressure against conductance of the four sensor-foam configurations; black: no foam; blue: foam A; pink: foam B; purple: both foams with sensor sandwiched between.

This section evaluates the response of the sensors to different foam configurations, inherently connected to even or uneven pressure distribution. The more uneven the pressure distribution, the more the conductance at peak load is expected to change. In fact, the pressure–conductance data of the sensor loaded without foam layer(s) were different from those after including the foams (Figure 3). At the same pressure levels, the conductance of the sensor was lower when loaded without foams. In any configuration, the sensor saturated at different conductance. The configuration without foams saturated at 18.98 mS; with foam A at 19.79 mS; with foam B at 21.06 mS; and with both foams at 23.15 mS. The saturation criterion was set to 0.4 MPa/mS (an increase in conductance of 1 mS would increase the pressure by 0.4 MPa). Although saturation is a gradual process, a criterion value was required for purposes of comparability. The pressure level at the saturation criterion was around 0.29–0.3 MPa. Note that the maximum average pressure reported by Ahmad et al. [10] was 0.11 ± 0.02 MPa.

In all four configurations, there was a crossover of loading and unloading curves (Figure 3), identified in foam B at 15.2 mS; in foam A at 10.45 mS; in the double foam configuration at 10.35 mS; and without foam at 7.4 mS.

The double foam configuration resulted in the most pronounced hysteresis behavior (Figure 3), followed by foam B, foam A, and finally the configuration without foams.

In summary, adding foams to the piezoresistive sensor used in this research severely affected its electrical behavior: the sensor became more conductive and more hysteretic as the thickness of the foam increased. The reason for this is that the thicker the foam, the more even the pressure distribution, and even more so if there are foams on both sides of the sensor. It is known [26] (Fuss et al., 2021, unpublished data) that unequal loading of piezoresistive pressure sensors with non-linear calibration curves affects the conductance of a sensor. The greater the thickness and the number of foams, the more the conductance changes, as seen in Figure 3. Therefore, despite the pronounced hysteresis, configuration 4 (sensor sandwiched between both foams) was selected for sensor calibration and subsequent pressure measurements. Furthermore, the sensor characteristics shown in Figure 3 are specific only to loading the sensor with the test conditions described in Section 2 (triangular displacement function with a specific strain rate). The increase in conductance as a function of the thickness of the foam layer is explained in Section 4.

### 3.3. Sensor Calibration and Validation

**Figure 4:**
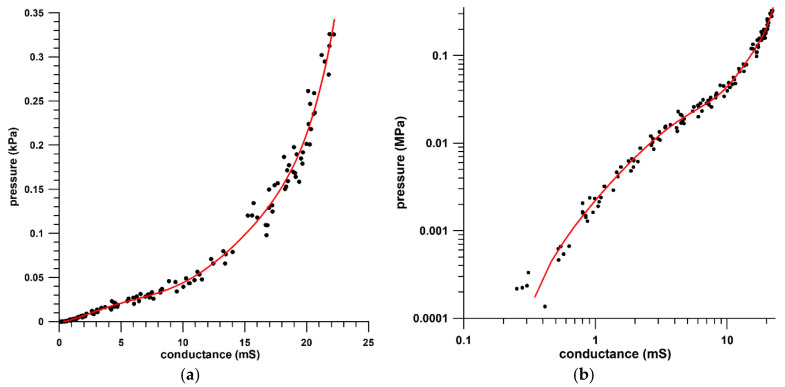
Pressure vs. conductance of the peak data, on Cartesian (**a**) and double-logarithmic (**b**) coordinate systems; the 6th-order polynomial fit line is indicated by the red curve. Although the data are the same in subfigures (**a**,**b**), the reason for displaying the data on Cartesian (**a**) and double-logarithmic (**b**) coordinate systems is that the Cartesian coordinate system magnifies large numbers whereas the double-log coordinate systems magnifies small data. To assess the goodness of fit of the function visually across all data, both displays are required.

The peak pressure and conductance data, obtained from the procedure described in Section 2, ranged from 0.136 MPa to 0.326 MPa, and from 0.10 mS to 22.19 mS. The data were fitted with a 6th-order polynomial to establish the calibration curve (Figure 4a,b):

*p* = 0.0000000465 *G*^6^ − 0.0000026800 *G*^5^ + 0.0000584227 *G*^4^ − 0.0005616387 *G*^3^ + 0.0023639815 *G*^2^ + 0.0006952569 *G* − 0.0003273922

for a 40 × 40 mm effective sensor area, where *p* is the pressure (unit: MPa) and *G* is the conductance (unit: mS). The R^2^ value of the calibration curve was 0.9873 and the RMSE was 0.0099 MPa. The polynomial was able to fit the data at both very small and high conductances, as shown in Figure 4a,b.

In contrast to the sensor evaluation results (Figure 3) the sensor did not saturate because it did not reach the saturation criterion of 0.4 MPa/mS at 0.3 MPa. At 22 mS, the pressure derived from the calibration curve was 0.3251 MPa, and d*p*/d*G* was 0.0764 MPa/mS.

**Figure 5:**
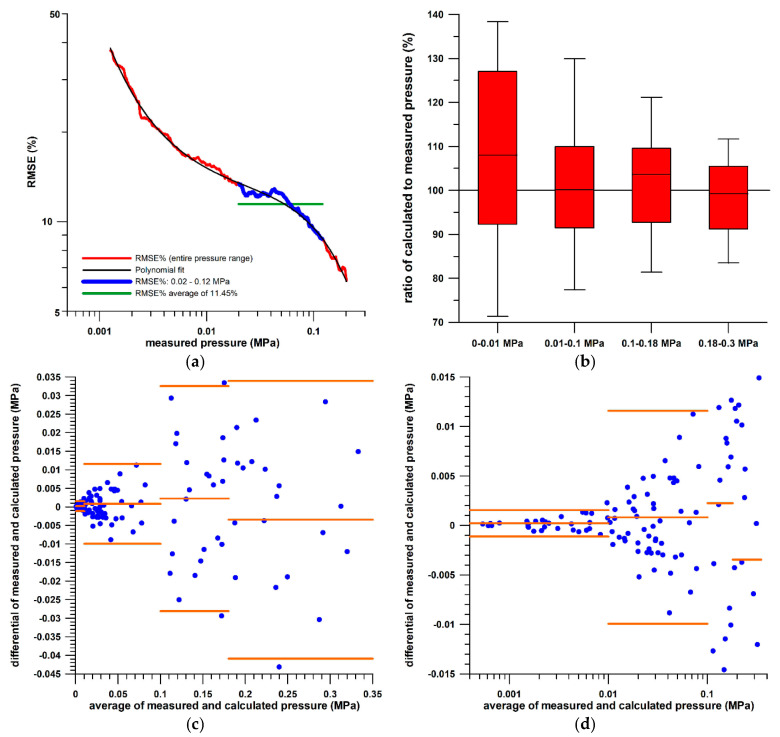
(**a**) Root mean square error (RMSE%) of the calculated pressure (as a percentage of the measured pressure) vs. the measured pressure; (**b**) box and whisker plot of the ratio of the calculated to the measured pressure (expressed as a percentage) for four different pressure ranges; (**c**,**d**): Bland–Altman plots of the pressure differential (measured pressure minus calculated pressure) vs. the average of measured and calculated pressures (linear and logarithmic scale); the orange horizontal lines indicate the average and the two limits of average ±1.96 standard deviations (95% confidence).

The peak data generated for the calibration exercise were used to validate the sensor. The calibration function obtained was applied to predicting the pressure from the measured conductance. The RMSE of the pressure calculated from the calibration function with respect to the force (converted to pressure) obtained from the force plate was approximately 21% at small pressures and decreased to approximately 7% at greater pressures (Figure 5a). The same behaviour is expressed by the histogram plots of the ratio of calculated to measured pressure (Figure 5b). The Bland–Altman plots (Figure 5c,d) revealed that the average pressure differences between measured and calculated pressure deviated from 0 only slightly over the four different pressure ranges, and that the individual pressure differences were almost entirely within the limits of average ± 1.96 standard deviations (95% confidence). The required clinical pressure range was 0.02–0.12 MPa according to the measurement shown in Section 3, which corresponds to an RMSE of 11.45% on average (Figure 5a).

### 3.4. Pressure Measurements

#### 3.4.1. Sensor Testing

**Figure 6:**
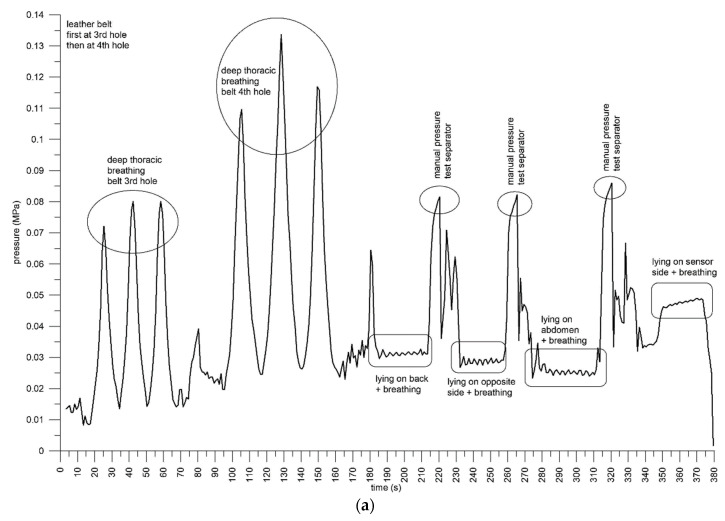

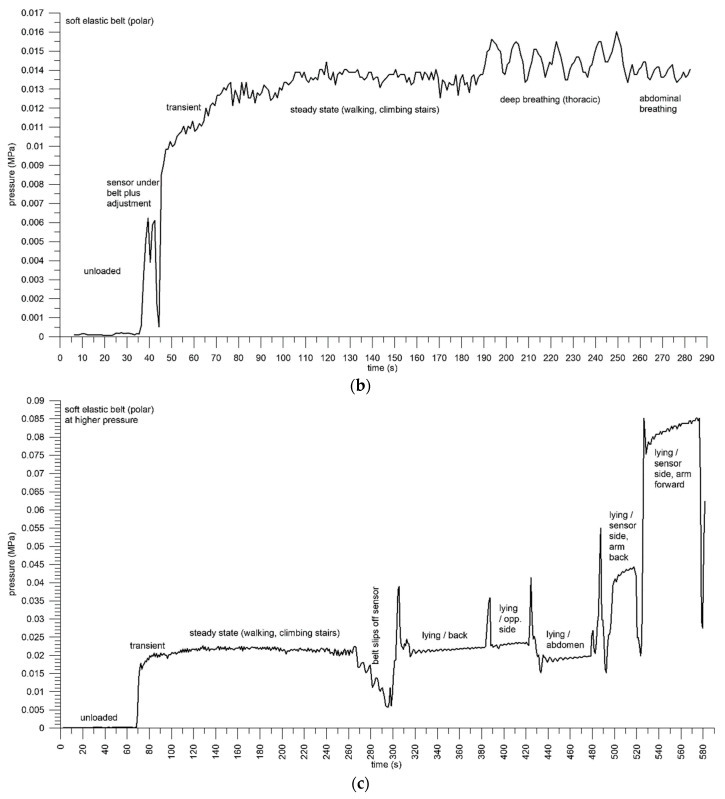

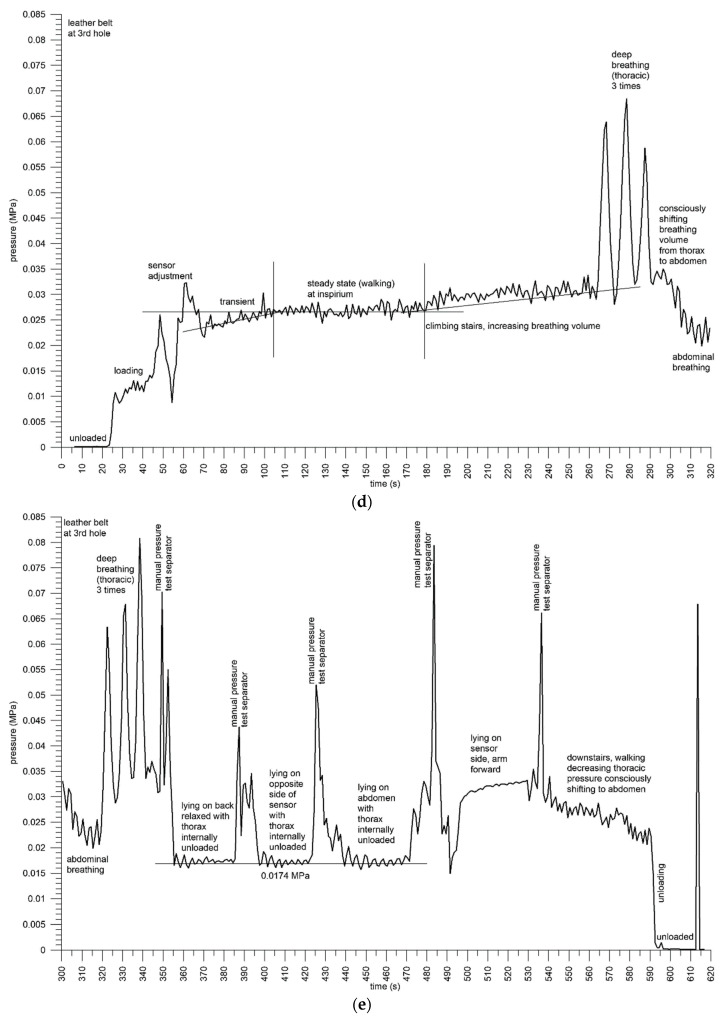
Pressure against time for Test 1 (**a**), Test 2 (**b**), Test 3 (**c**) and Test 4, first (**d**) and second (**e**) halves, respectively.

Test 1: The pressure was applied with a leather belt, with the buckle at the 3rd and then at the 4th hole (from the end of the belt) when standing. The reason for tightening the belt was to verify that belt tensioning can be detected. In fact, the pressure increased. The baseline data were at 0.015 and 0.025 MPa at the 3rd and 4th holes, respectively, and the pressure increased at deep inspiration to 0.07–0.08 MPa and 0.11–0.13 MPa at the 3rd and 4th holes, respectively (Figure 6a). Lying in different positions produced pressures from 0.03 to 0.05 MPa when the belt was taut at the 4th hole. The pressure when lying on the back, abdomen, and on the opposite sensor side was comparable to the baseline pressure when standing (0.015–0.025 MPa). Lying on the sensor side increased the pressure, through external forces acting on the sensor, to 0.05 MPa.

Test 2: A softer, elastic belt (A300, Polar, Kempele, Finland) expectedly resulted in smaller pressure values, around 0.013–0.016 MPa (Figure 6b). When adjusting the belt, the pressure increased from 0 to 0.01 MPa, followed by a short transient phase, and a subsequent steady-state signal with consistent pressure values. The average was 0.0135 MPa when walking and climbing stairs. This pressure average remained as a baseline value when deep breathing. The peak data due to deep abdominal breathing were evidently smaller than when breathing thoracically, because the sensor was attached to the lateral thorax.

Test 3: Increasing the pressure with the elastic belt (A300, Polar, Kempele, Finland) resulted in greater pressure values, initially at 0.015 MPa and after the transient at 0.02 MPa, with a steady state when walking and climbing stairs (Figure 6c). After 260 s, the belt slipped off the sensor gradually, and the test person moved the belt back onto the sensor before lying on the back, abdomen, and opposite sensor side. The same result seen in Test 1 was verified in this test, namely, that the pressure when lying in these three positions was comparable to the baseline pressure when walking (approximately 0.02 MPa). In this test, lying on the sensor side was performed with two different arm positions: when retroverting the arm, more pressure was shared with the sensor and thus the sensor pressure was only 0.04–0.045 MPa, whereas when anteverting and elevating the arm, the sensor pressure increased to 0.08–0.09 MPa (Figure 6c).

Test 4, with the leather belt at the 3rd hole, was conducted over 10 min (Figure 6d,e). After tightening and adjusting the belt, and after a short transient period, the pressure remained constant at 0.025–0.03 MPa, when walking and breathing at the inspirium. When climbing stairs, the pressure increased slightly to 0.03 MPa. Subsequent deep thoracic breathing produced peak pressures at around 0.06–0.07 MPa, at a baseline of 0.03–0.035 MPa. Shifting the breathing consciously from thoracic to abdominal reduced the baseline to 0.02–0.025 MPa. Further deep thoracic breathing returned to peak values of 0.065–0.08 MPa. Lying relaxed on the back, abdomen, and opposite sensor side produced a constant pressure of 0.0174 MPa despite turning. Lying on the sensor side increased the pressure to 0.03–0.035 MPa. When walking downstairs, and thereby shifting the breathing from thoracic to abdominal, the pressure decreased by approximately 0.01 MPa.

In summary, the sensor responded well to different conditions, so the sensor was subsequently tested inside a scoliosis brace.

#### 3.4.2. Sensor Application

**Figure 7:**
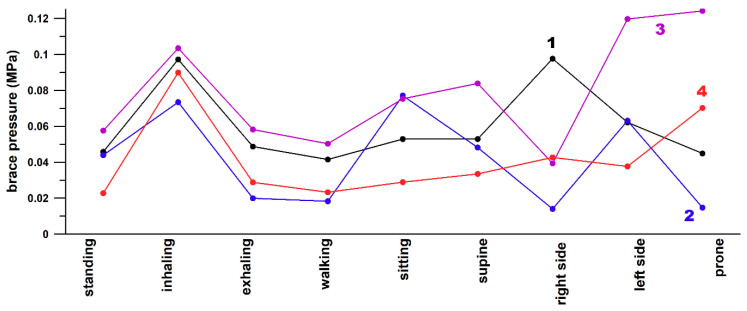
Average sensor pressure data recorded inside the brace at different body positions, and at different levels of the scoliosis curve (thoracic and thoracolumbar); 1: participant A, thoracic curve; 2: participant A, thoracolumbar curve; 3: participant B, thoracolumbar curve; 4: participant B, thoracic curve.

We measured the pressure inside the Chêneau scoliosis braces (Figure 1b) and obtained pressure data over the range of 0.014–0.124 MPa (Figure 7). On average, the maximum pressure was generated when inhaling (0.91 MPa), and the minimum pressures when exhaling and walking (0.039 and 0.033 MPa, respectively).

The 36 pairs of positions exhibited a significant pressure difference (p < 0.05) in most positions, except for: standing/exhaling, standing/right, exhaling/walking, sitting/supine, sitting/prone, supine/right, left/prone with insignificant differences in all four post-hoc tests (p > 0.05), and standing/walking and sitting/right with insignificant differences only in the Scheffé post-hoc test.

The difference in pressure between thoracic and thoraco-lumbar curves was not significant (p > 0.05) while exhaling and walking. In the significant pressure differences (p < 0.05), the thoraco-lumbar pressure was higher than the thoracic pressure in all other positions, except for inhaling, where the thoracic pressure was evidently higher, and for right and left positions, where the pressure depends on which side the curves and the sensor were located.

## 4. Discussion

### 4.1. The Calibration Problem and the Sensor Accuracy

It was known from previous research [25] that sensors loaded in series with viscous solids become more viscous with greater hysteresis. It was therefore expected that different foam padding would affect the electrical behavior of the sensor. This phenomenon was verified with a standardized method (Figure 3).

Why wasn’t the calibration function derived from the data shown in Figure 3? Pressure versus conductance data with pronounced hysteresis offer several options for attempted calibration. The first four options refer to the calibration curve obtained from loading and unloading the sensors with a material testing machine (Figure 3); the fifth option refers to manual loading of the sensor (Figure 4a,b):

(1) using the loading segment only;
(2) using the unloading segment only;
(3) using the average of loading and unloading segments at any specific conductance value;
(4) using both loading and unloading segments and applying them depending on the sign function of the pressure rate (loading if positive pressure rate and vice versa);
(5) using only peak data for calibration.

**Figure 8:**
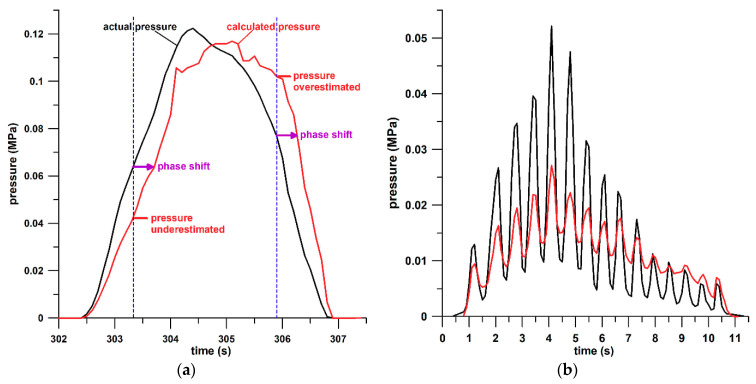
Pressure obtained from the force plate data (black) and from the sensor (red; calculated from the calibration curve) against time. (**a**) The inaccuracies resulting from using peak data for calibration can be seen as inaccuracies of magnitude (under- and overestimation of the pressure data) at the correct time, or as time inaccuracies of accurate pressure affected by a phase shift (slight delay); the indicated phase shift (purple arrow) corresponds to approximately 0.4 s. (**b**) Fast pressure changes (approx. 1.5 Hz loading cycles) exhibit the damping effect (reduced peak data) caused by the sensor structure (viscous structure of sensor plus foams).

All options have advantages and disadvantages; however, the best solution to this problem is using the peak data only for establishing the calibration curve (Figure 4a,b). In this case, it is evident that the pressure rise and fall data will suffer from inaccuracies. However, as the sensor hysteresis is a viscous and thus a time-dependent behavior, the apparent pressure inaccuracies of the rise and fall data can be seen as a phase-shift, i.e., the accurate pressure occurs at the wrong time, so that a pressure RMSE assessment of rise and fall segments is no longer relevant and therefore not applicable. When considering that peak data occur at the transition from pressure rise (loading) to fall (unloading), then the master calibration curve is located between the loading curve and the unloading curve. At the same conductance, the pressure data of the loading curve are usually higher than those of the unloading curve. When using the master calibration curve, pressure data during loading are underestimated (because the master calibration curve delivers smaller pressures than the loading curve at the same conductance), whereas the pressure data during unloading are overestimated (Figure 8a). Rather than criticizing these pressure inaccuracies, the pressure data can be considered to be accurate but occur at the wrong time, that is, slightly delayed by a phase shift (Figure 8a) or phase inclination.

Independent of the choice, after establishing a calibration function, the sensor has to be validated. Additional structures placed in series with a sensor, such as foams and other viscous materials, make the sensor highly dependent on the test conditions. It is therefore advantageous to establish the calibration function, and validate the sensor, under, or close to, the conditions of the sensor’s ultimate application. This was verified by means of showing the difference between the pressure–conductance relationship of test condition 4 (sensor plus two foams) tested with standardized triangular displacement and the actual calibration function derived from force-plate testing.

If the sensor is loaded with conditions other than those of the sensor’s ultimate application, then the calibration function fails to return the correct pressure values. A typical example is shown in Figure 8b, where the sensor was loaded at a higher frequency. The calculated pressure data appear to be affected by an “electrical” damping effect, which is expected because foams are ideally suited for this specific application (mechanical damping and energy absorption).

The shortcomings of the sensor developed and evaluated in this research are caused by the foams placed next to the sensor, rather than by the sensor itself.

Although the RSME data of the rise and fall segments are no longer relevant, as explained above, the RMSE% of the peak data is still applicable. Within the clinically relevant pressure range, the RMSE% was 11.45 ± 1.32%. This is a typical value for piezoresistive pressure sensors. For example, Weizman et al. [24] reported similar values in two different smart insoles, ranging from 5.6–14.6% and 11.2–13.2%, respectively.

### 4.2. Foam Properties Affecting the Conductance of the Sensor

In sensors with a non-linear pressure–conductance curve, as shown in Figure 3, unequal loading causes a change of the sensor conductance (Fuss et al., 2021, unpublished data). Because foams facilitate a more even pressure distribution, it is expected that the conductance will change if the sensor is loaded in series with a piece of foam. It appears that the conductance increases as the foam thickness increases, however, the pressure is more evenly distributed in softer foams. The softest foam is the combination of foams A and B, followed by foam B. However, foam B produces the second lowest conductance of the sensor. This is because a softer foam bottoms out faster, i.e., reaches the densification regime faster, which in turn increases the stiffness of the foam at greater loads. At 0.12 MPa, both foams are compressed at approximately 6.5% strain: foam A to the beginning of the collapse phase, and foam B halfway through the linear elastic phase.

### 4.3. Brace Pressure Data

Wong and Evans [7] measured the pressure inside the brace with an electrohydraulic pressure-sensing system, whose measurement range ended at 240 mmHg (0.032 MPa) which, according to Wong and Evans [7], seemed “adequate for the measurement purpose”. In fact, the maximum pressure measured (according to the graphs of [7]) was slightly under 200 mmHg (0.027 MPa).

The findings by Ahmad et al. [10] were “similar to the studies” published by Wong and Evans [7] and Pham et al. [9] in terms of significant differences. However, the pressure data reported by Pham et al. [9] (0.0073 ± 0.00064 MPa (“before adjustment” data)) and Wong and Evans [7] (70 ± 10 mmHg = 0.0093 ± 0.0013 MPa on average) were surprisingly smaller by approximately the order of one magnitude compared to the data obtained by Ahmad et al. [10] (0.067 ± 0.013 MPa (“normal tension” data)) and to the data found in our study (Figure 7; 0.056 ± 0.029 MPa). The data reported by van den Hout et al. [8] were on average 0.043 ± 0.005 MPa on the lumbar sensor and 0.015 ± 0.004 MPa on the thoracic sensor, which ranged between the aforementioned low- and high-pressure data. There are several possible explanations for the source of these huge differences in pressure, and Wong and Evans [7] and Pham et al. [9] reported similar data, in addition to Ahmad et al. [10] and the current study. Firstly, these studies used different TLSOs, namely the Milwaukee, Boston, and Chêneau braces; the difference in design could lead to variation of the inside brace pressures. Secondly, these studies used different pressure measurement systems. Finally, as these studies spanned a period of 20 years, different strap tensions could have been the reason for the results; if so, then the recommendations for strap tension forces could have changed over that period.

## 5. Conclusions

Although the hard-shell TLSO brace has existed since the early 1970s, after nearly 50 years there is still no consensus on the required magnitude of corrective pressure for ideal therapeutic results. One of the main hurdles have been the cost and bulkiness of the currently available sensors to measure brace pressure for long periods. The results from this study hope to pave the way for a cheaper, more mobile system to be used for future longitudinal studies on comfort and pressure measurement of braces during daily activities. Such studies can be conducted by correlating, e.g., the average pressure impulse calculated over a longer time to the change of the Cobb angle across the same period. Reducing the Cobb angle, or at least preventing worsening of the Cobb angle, would be the desired result. Furthermore, the compliance of the patients can also be assessed.

The foam liners increased the hysteresis of the sensor because foams are both viscous and energy absorbers. The hysteresis of a calibration curve, resulting in different pressure levels for loading and unloading at the same conductance (or different conductances at the same pressure level), has two alternative effects. Either the magnitude of the calculated pressure is considered to be inaccurate (under- and over-estimation of the pressure during loading and unloading, respectively); or the timestamp of the pressure data is considered to be inaccurate, because the pressure data are slightly delayed by the phase shift of the calculated pressure with respect to the applied pressure.

The piezoelectric pressure sensor developed in this study proved to be both accurate and cost effective for a clinical trial because it reacts to the changing pressure during breathing and different activities.

## Figures and Tables

**Table 1:** Comparison of energy absorption per unit volume (*W*), strain (*ε*), and stress (*σ*) of foams A and B.

Properties	Average Data of Foam B (Pink)	Average Data of Foam A (Blue)	Ratio (B/A)	Comments
maximum *W*/*σ* (-)	0.2948	0.3066	0.9615	same
*ε* (-) at maximum *W*/*σ*	0.5496	0.5596	0.9822	same
maximum *E* (MPa) at linear elastic segment	7.1613	3.6154	1.9808	B = twice as stiff
minimum *E* (MPa) at the collapse plateau	0.3655	0.1457	2.5082	B = 2.5 times as stiff
thickness (mm)	6.15	3.17	1.9400	B = twice as thick
maximum *F* (N)	5000	2500	2	depends on test conditions, *F*_B_ = 2 *F*_A_
maximum *σ* (MPa) at maximum *F*	2.0382	1.0407	1.9585	depends on test conditions
maximum *W* (kJ/m^3^) at maximum *F*	443.457	245.416	1.8070	depends on test conditions
maximum *ε* (-) at maximum *F*	0.7411	0.7571	0.9789	same; depends on test conditions and on stiffness (foam B is approximately twice as stiff as foam A)
Strain rate (s^−1^)	0.032	0.032	1	=test condition

## Data Availability

The data presented in this study are available on request from the corresponding author to any qualified researcher, if they have obtained Ethics Approval for secondary use of existing data through a Consent Waiver.
